# Fast, Potent Pharmacological Expansion of Endogenous Hes3+/Sox2+ Cells in the Adult Mouse and Rat Hippocampus

**DOI:** 10.1371/journal.pone.0051630

**Published:** 2012-12-10

**Authors:** Simone Pacioni, Maria Adele Rueger, Giuseppe Nisticò, Stefan R. Bornstein, Deric M. Park, Ron D. McKay, Andreas Androutsellis-Theotokis

**Affiliations:** 1 European Brain Research Institute, Rome, Italy; 2 Department of Neurology, University of Cologne, Cologne, Germany; 3 Department of Medicine, University of Dresden, Dresden, Germany; 4 Department of Neurological Surgery, University of Virginia, Charlottesville, Virginia, United States of America; 5 Lieber Institute for Brain Development, Baltimore, Maryland, United States of America; 6 Center for Regenerative Therapies Dresden, Dresden, Germany; University of Nebraska Medical Center, United States of America

## Abstract

The adult hippocampus is involved in learning and memory. As a consequence, it is a brain region of remarkable plasticity. This plasticity exhibits itself both as cellular changes and neurogenesis. For neurogenesis to occur, a population of local stem cells and progenitor cells is maintained in the adult brain and these are able to proliferate and differentiate into neurons which contribute to the hippocampal circuitry. There is much interest in understanding the role of immature cells in the hippocampus, in relation to learning and memory. Methods and mechanisms that increase the numbers of these cells will be valuable in this research field. We show here that single injections of soluble factors into the lateral ventricle of adult rats and mice induces the rapid (within one week) increase in the number of putative stem cells/progenitor cells in the hippocampus. The established progenitor marker Sox2 together with the more recently established marker Hes3, were used to quantify the manipulation of the Sox2/Hes3 double-positive cell population. We report that in both adult rodent species, Sox2+/Hes3+ cell numbers can be increased within one week. The most prominent increase was observed in the hilus of the dentate gyrus. This study presents a fast, pharmacological method to manipulate the numbers of endogenous putative stem cells/progenitor cells. This method may be easily modified to alter the degree of activation (e.g. by the use of osmotic pumps for delivery, or by repeat injections through implanted cannulas), in order to be best adapted to different paradigms of research (neurodegenerative disease, neuroprotection, learning, memory, plasticity, etc).

## Introduction

The involvement of immature cells (neural stem cells and neural progenitor cells) in the normal function and repair capacity of the adult brain is becoming increasingly appreciated. Since the initial observations that new neurons can be generated in certain areas of the adult mammalian brain [1], [2], populations of stem cells have been described in various brain and spinal cord regions [1], [3]–[23]. It was soon recognized that insults such as ischemic stroke and epilepsy were able to mobilize these endogenous cells, suggesting their readiness to respond to various challenges [15], [19], [22], [24], [25], [26]. Enriched environments and physical exercise are also able to promote adult neurogenesis [1], [27]. Additional work has identified genes that are prominently expressed in these cells and help with their identification [3], [6], [28]–[46]. Culture systems and in vivo validation experiments are generating strategies for the manipulation of these cells in situ [6], [8], [43], [47]–[61]. The aim, in many of these approaches, is the replacement of lost cells from endogenous sources. However, a more recent strategy aims to stimulate endogenous stem cells and induce them to provide trophic support to injured neurons; in this way, endogenous neural stem cells are used as mediators or neuronal rescue.

**Figure 1:**
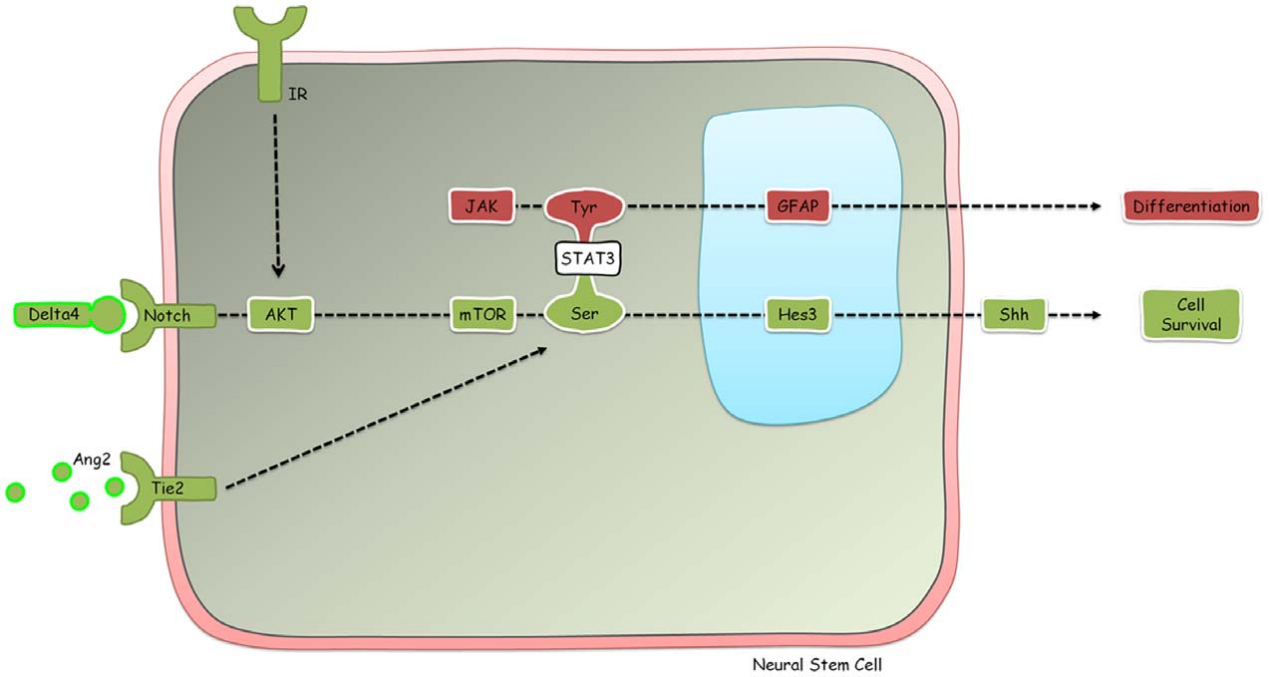
A signaling pathway that regulates the numbers of neural stem cells *in vitro* and *in vivo*. Different STAT3 phosphorylation states affect different properties of neural stem cells: serine phosphorylation induces survival whereas tyrosine phosphorylation induces differentiation. STAT3-serine phosphorylation can be induced by ligands of the Notch and Tie2 receptors, both of which are produced by vascular endothelial cells. Insulin also induces STAT3-serine phosphorylation. STAT3-tyrosine phosphorylation is induced by the JAK kinase. For a more detailed analysis of the signaling, please see Masjkur et al (In Press).

We have previously elucidated a signal transduction pathway that regulates the numbers of neural stem cells in vitro and in vivo [42], [43], [48], [62], [63] (Figure 1). Inputs to this pathway include a non-canonical branch of the Notch signaling pathway (which can be activated by treatment with the soluble ligands Delta4 and Jagged1), activation of the Tie2 receptor by Angiopoietin 2, insulin, and the established mitogen of neural stem cells basic Fibroblast Growth Factor (bFGF). At the convergence point of these inputs is the phosphorylation of the signaling molecule STAT3 on the serine residue. Because phosphorylation of STAT3 on the tyrosine residue induces the differentiation of neural stem cells to the astroglial fate [64], [65], [66], [67], [68], it is critical that in order to expand neural stem cell numbers via STAT3-serine phosphorylation, treatments must not induce STAT3-tyrosine phosphorylation. Treatments that induce STAT3-serine phosphorylation in the absence of STAT3-tyrosine phosphorylation also induce the transcription of the transcription factor Hairy and Enhancer of Split 3 (Hes3) [42]. Hes3 is expressed in the developing brain where it regulates the timing of the differentiation of the neural precursor population [69], [70]. We have reported that Hes3 expression persists in the adult, where it identifies putative neural stem cell populations in many areas of the brain [43]. In fact, established fetal and adult rodent neural stem cell cultures express Hes3 and Hes3 expression is lost following induced differentiation.

Hes3 null mice show a generally normal phenotype. Neural stem cell cultures from adult Hes3 null mice can be established, suggesting that the role of Hes3 is not essential for their maintenance [48]. However, Hes3 null cultures exhibit a much reduced response to treatments with Delta4 and insulin. These results show that Hes3 mediates functions that are directly relevant to the regenerative response after injury. In this work, we focused on Hes3 expression in order to measure the effects of treatments on the putative endogenous neural stem cell/progenitor cell population in the adult mouse and rat brains. In the adult brain, Delta4 and Angiopoietin 2 (Ang2) are produced by vascular endothelial cells, with which Hes3+ cells physically associate, suggesting that the activation of Hes3+ cells by the exogenous factors used here may reflect a natural regenerative response of the adult brain to injury [71].

Our previous work has presented a therapeutic strategy that relies on the neuroprotective effects of endogenous neural stem cells, presumably through their trophic support on injured neurons [42], [43], [48]. There is no evidence that neuronal replacement is involved in this process. Here we show that soluble factors can also increase the numbers of Hes3+ cells in the adult rodent hippocampus, a neurogenic zone of the adult brain. Future work may reveal if in this area, neurogenesis as well as learning and memory functions can also be affected by our treatments.

## Results

Our experimental approach has been to perform single injections of soluble factors in the lateral ventricles of adult rats and mice, and to assess changes in the numbers of putative stem cells/progenitor cells 5 days (rat) or one week (mice) later. In the case of the rats, we used a combination treatment which we have used in the past and which we had already shown to have potent effects in other brain areas [43]. This combination treatment consists of the Notch receptor ligand Delta4, the Tie2 receptor ligand Angiopoietin 2, insulin, and an inhibitor of the JAK kinase. We used this combined treatment because we have shown that (a) it is a powerful activator of endogenous neural stem cells in many brain areas and (b) it does not have significant adverse effect on the vasculature, in contrast to Delta4, Angiopoietin 2, and insulin, when administered alone.

For the mouse experiments, we used a simplified combination treatment consisting of Delta4 and Angiopoietin 2. This was in order to select one pro-angiogenic factor (Angiopoietin 2) and one anti-angiogenic factor (Delta4), both of which alone or in combination increase the numbers of endogenous Hes3+ cells. Therefore, we show that a simplified pharmacological treatment, relative to our previous work, is able to efficiently increase the numbers of Hes3+ cells in the adult brain. Hes3+ cells in the adult rat represent a subpopulation of cells expressing the neural progenitor marker Sox2 [38] and here we used double immunostaining for Hes3 and Sox2 to measure effects of treatments. We focus on a brain area which we have not looked in before, the hippocampus. These observations extend the relevance of our previous work to the fields of neurodegenerative diseases affecting the hippocampus, learning, and memory.

### Pharmacological induction of proliferation in the adult rat hippocampus by a combination of soluble factors

**Figure 2:**
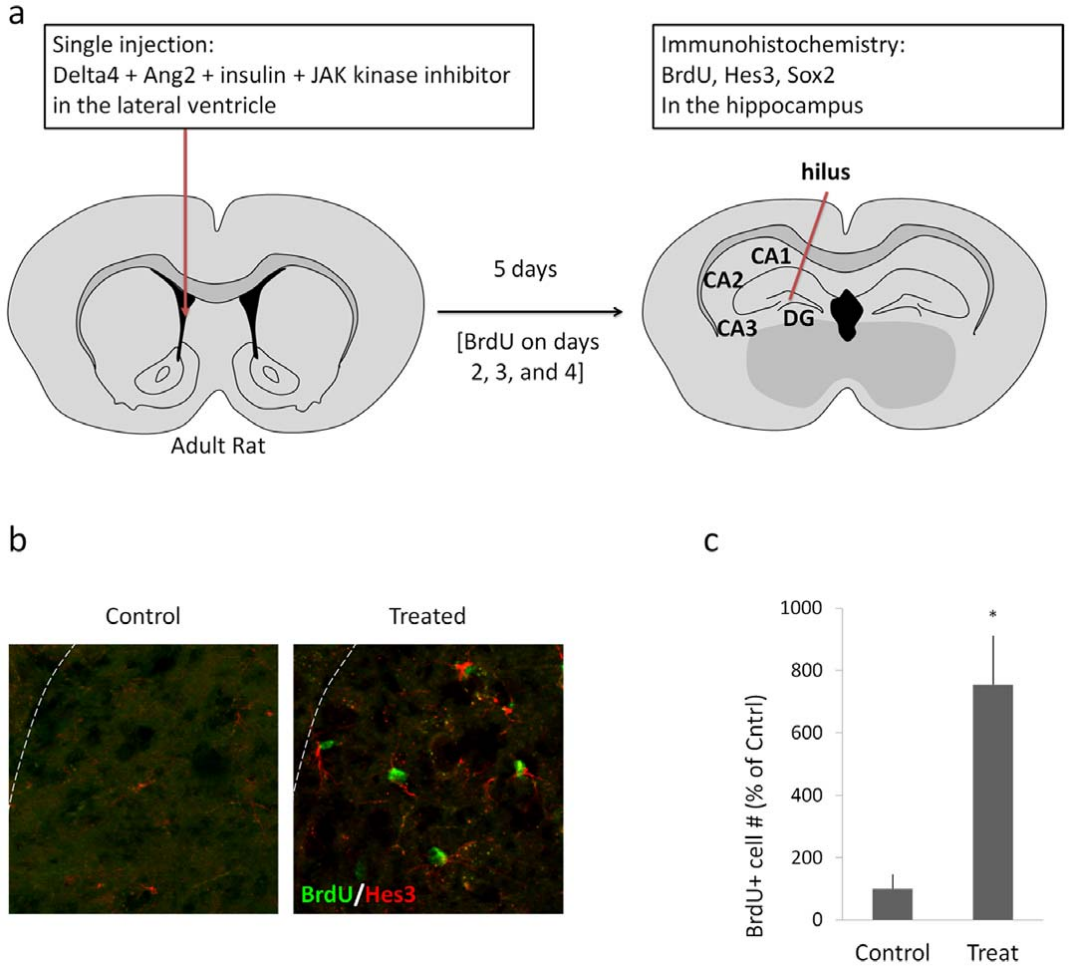
Soluble factors induce cell proliferation in the adult rat hippocampus. (a) A schematic diagram shows the site of a single injection (lateral ventricle) of soluble factors (a combination of Delta4, Angiopoietin 2, insulin, and a JAK kinase inhibitor) in adult rats. The day of injection was termed Day 1. On days 2, 3, and 4, intraperitoneal BrdU injections were administered as described in the Materials and Methods section. On Day 5, animals were perfused and the brains were prepared for immunohistochemical analysis. The data presented are from the hilus of the hippocampus where the major effects were observed. (b) Immunohistochemical detection for BrdU and Hes3 in the hilus of control and treated animals shows BrdU/Hes3 double-positive cells. (c) Quantification of the effect of the treatment on the number of BrdU labeled cells in the hilus. For the graph, the number of cells in the sham operated control animals was set to 100%. Treatments induced a several-fold increase (p<0.05). [Width of images: b, 325 micrometers].

In the experiments involving adult rats (N = 7 per each group), a single injection of a combination of Delta4, Angiopoietin 2, insulin, and a JAK kinase inhibitor, dissolved in saline, was performed into the right lateral ventricle, as described in the Materials and Methods section (Five microliters of a solution containing: Delta4, 2 mg/mL; Ang2, 1 mg/mL; insulin, 8 mg/mL; Jak Inhibitor, 20 μM). Vehicle controls were injected with only saline. The day of injection was noted as day 1. On days 2, 3, and 4, intraperitoneal injections of BrdU were performed (2 per day) in order to label dividing cells. On day 5, the rats were perfused in order to collect the brains and process them for immunohistochemical analysis. Figure 2a provides a schematic diagram summarizing the experimental procedure. In the experiments with rats, we focused our analyses specifically on the hilus of the dentate gyrus because we observed vast changes due to our treatment. Numbers are expressed as a percentage of the number of cells per field of view in the controls [I.e. control values are set to 100% +/− standard deviation].

The treatment induced a 7,54-fold increase in the number of BrdU-labeled cells in the hilus of the dentate gyrus (Vehicle control: 100% +/−47; Treatment: 754% +/−158; p<0.05) (Fig. 2b,c).

**Figure 3:**
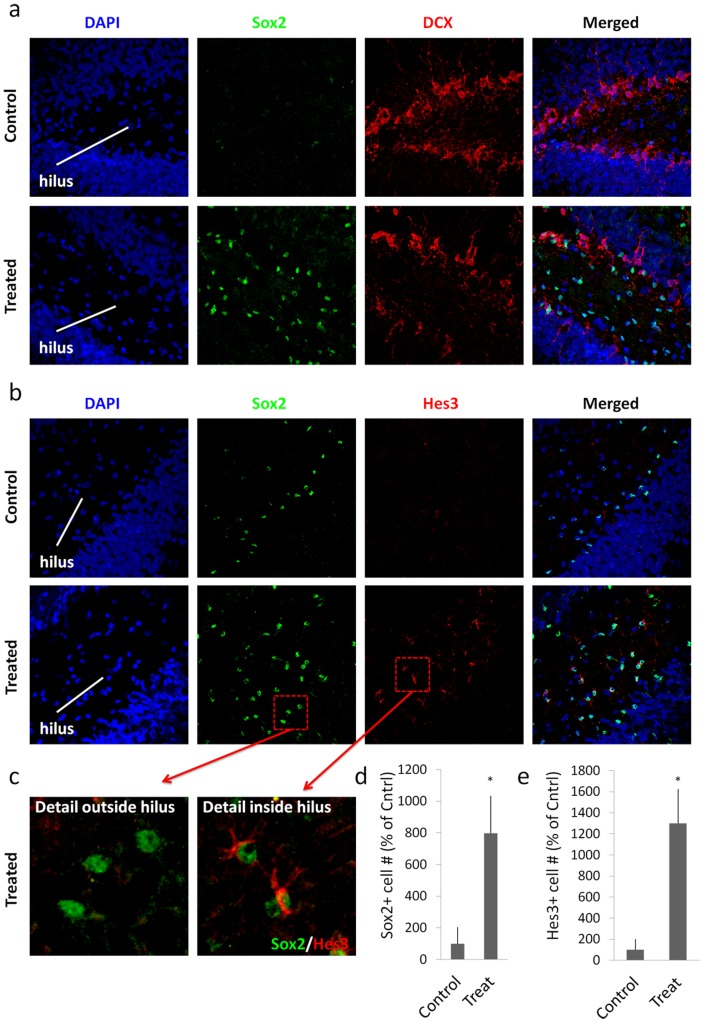
Soluble factors increase the numbers of Hes3+ cells in the adult rat hippocampus. (a) Treatment with the soluble factors induced a powerful increase in the number of Sox2+ cells in the hilus [Quantifications are from images taken from the tip of the hilus to approximately 1,300 micrometers towards the CA3 region – representative images are shown. Staining for the young neuron marker doublecortin (DCX) helps to visualize the subgranular zone]. (b) The same treatment also induced an increase in the numbers of Hes3+ cells in the hilus. (c) Sox2+ cells inside the hilus co-expressed Hes3; in contrast, Sox2+ cells in the subgranular zone did not co-express Hes3. (d,e) Quantification graphs of the increases in Sox2+ and Hes3+ cell numbers in the hilus (p<0.05). For the graph, the number of cells in the sham operated control animals was set to 100%. [Width of images: a, 650 micrometers; b, 650 micrometers].

To corroborate the immature nature of these cells, we performed immunohistochemistry against the marker Sox2 [72], [73], [74]. Similarly to the BrdU results, we determined that the treatment also increased the numbers of Sox2+ cells in the hilus (Fig. 3a,b,c,d). Co-staining with the young neuron marker doublecortin (DCX) helps to visualize the subgranular zone. The increase in numbers was comparable between BrdU and Sox2 (Sox2: Control: 100% +/−34; Treatment: 798% +/−113; p<0.05).

The numbers of Hes3+ cells increased even more than those for BrdU and Sox2 (Fig. 3b,e). Very few Hes3+ cells were observed in the hilus of control (Vehicle-injected) rats (approx. 0–2 Hes3+ cells per hilus). The treatment induced a 13-fold increase in the number of Hes3+ cells (Control: 100% +/−100; Treatment: 1300% +/−324; p<0,05). All Hes3+ cells also expressed Sox2; this result, in combination with our previous work on Hes3+ cells suggests a putative stem cell/progenitor cell identity [42], [43], [48], [62], [63].

The most prominent changes observed in these experiments were inside the hilus. However, increased Sox2+ cell numbers were also observed outside the hilus, in the subgranular zone. Sox2+ cells in the hilus co-expressed Hes3. In contrast, Sox2+ cells outside the hilus and in the subgranular zone did not co-express Hes3 (Fig. 3b,c). This result suggests that Hes3 identifies a subpopulation of Sox2+ cells (or a particular state of these cells), which is prominent in the hilus. Fig. 3b,c shows low and high power examples of images obtained following immunohistochemical labeling for Sox2 and Hes3. Hes3 can be clearly seen to distinguish the Sox2+ cell subpopulations in and out of the hilus.

### Increases in Hes3+ cell numbers in the adult mouse hippocampus by a combination of soluble factors

**Figure 4:**
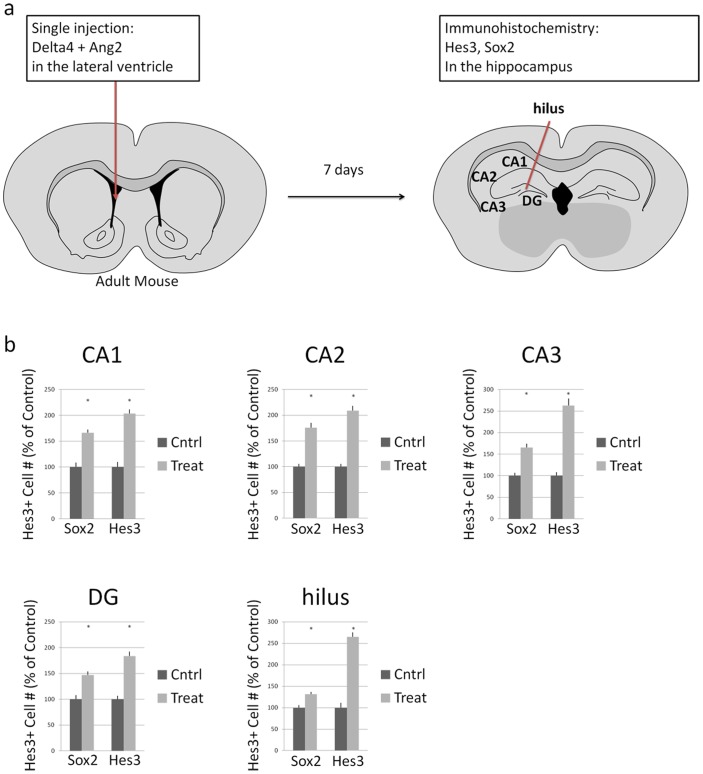
Soluble factors increase the numbers of Hes3+ cells in the adult mouse hippocampus. (a) A schematic diagram shows the site of a single injection (lateral ventricle) of soluble factors (a combination of Delta4 and Angiopoietin 2) in adult mice. Analysis was performed 7 days following the injection. (b) Quantification of the number of Sox2+ and Hes3+ cells in different areas of the hippocampus (numbers from control animals are shown as 100%).

**Figure 5:**
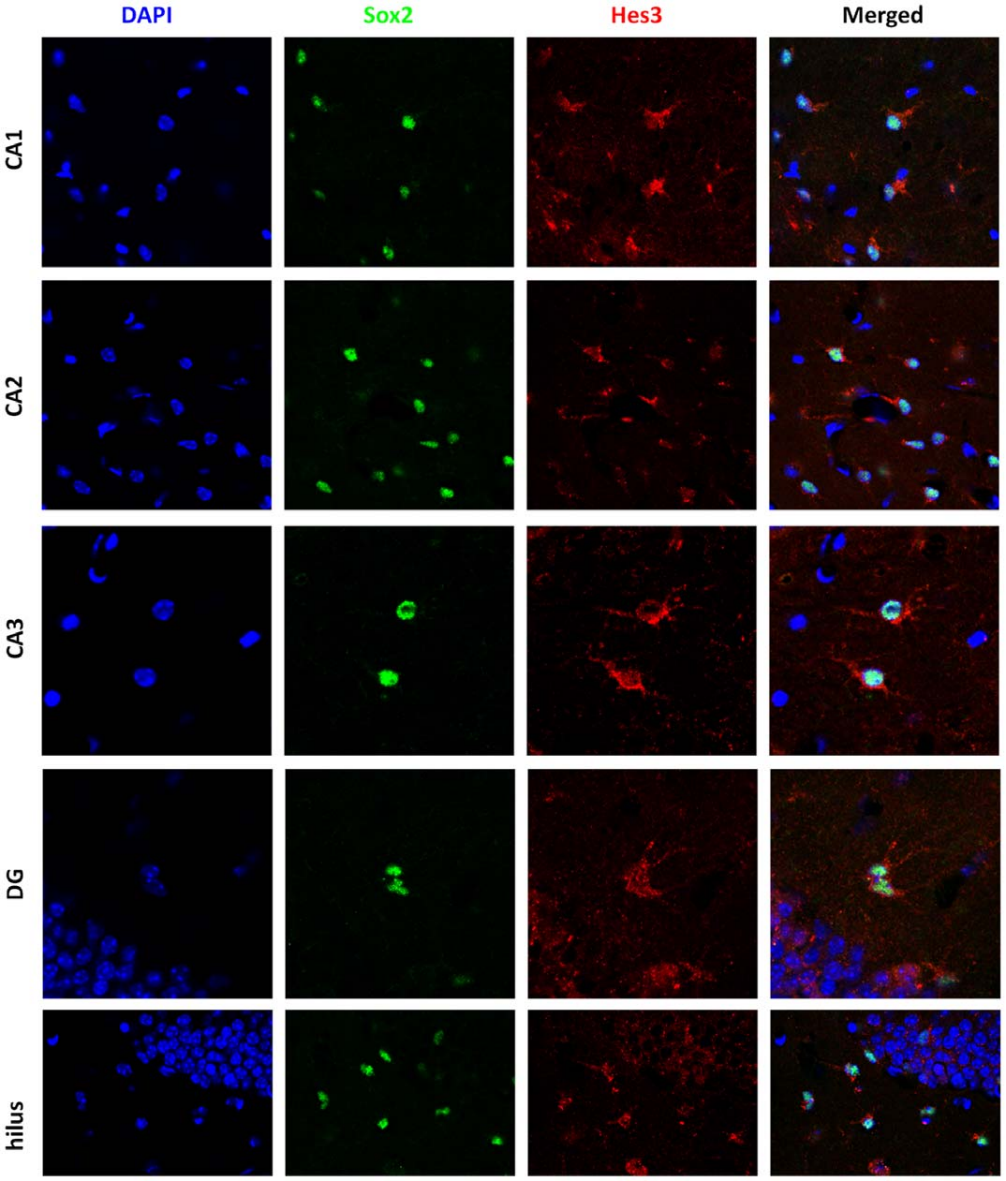
Soluble factors increase the numbers of Hes3+ cells in the adult mouse dentate gyrus and hilus. Representative images show Sox2+ and Hes3+ cells in various areas of the treated hippocampus. [Width of images: CA1, CA2, hilus: 115 micrometers; CA3, DG: 80 micrometers].

In the adult mouse, we performed a single intracerebroventricular injection of a combination of Delta4 and Angiopoietin 2 (Two microliters of a combination of: Delta4, 2 mg/mL; Ang2, 1 mg/mL), using stereotaxic coordinates, and we euthanized mice one week later for tissue analysis by immunohistochemistry. Figure 4a provides a schematic representation of the experimental design. Figure 4b provides quantifications of Sox2+ and Hes3+ cells in different regions of the hippocampus, under control (vehicle injection) and treatment conditions. Table S1 and Table S2 provide the numbers used for these graphs with statistical analysis data as well as fold-changes between control and experimental groups. Figure 5 provides immunohistochemical examples of the effects described. Figure S1 provides images from control (saline-injected) animals that can be used for comparison with Figure 5. Figure S2, Figure S3, and Figure S4 provide additional examples of the effects of pharmacological treatment on Sox2+ and Hes3+ cells. Here, we observed increases in Hes3+ cell numbers in all areas of the hippocampus within one week from a single intracerebroventricular injection of combined Delta4 and Angiopoietin 2. This result is in accordance with our previous work showing that treatment of cultured neural stem cells with Delta4 induces Hes3 transcription [42]. As we discuss later in the text, the strongest immunohoistichemical signal for Hes3 was observed in the dentate gyrus (and specifically, in the hilus and subgranular zone). These observations are further expanded in the Discussion section.

### CA1 region

In the CA1 region, the treatment induced a significant but relatively weak effect on the number of Sox2+ cells (Control: 100% +/−8.6, s.e.m.; Treatment: 166.1% +/−6.1, s.e.m.). The response of Hes3+ cells was greater (Control: 100% +/−9.7, s.e.m.; Treatment: 203.6% +/−7.8, s.e.m.) [s.e.m.: standard error of the mean]. Table S1 presents the data in absolute values; Table S2 presents the same data as fold increases. These results show significant increases in the numbers of Sox2+ and Hes3+ cell number following pharmacological treatment in the adult mouse CA1 region, and a greater effect on the Hes3+ subpopulation of Sox2+ cells. A recurring observation was the appearance of doublets of Sox2+/Hes3+ cells, suggesting these are the products of cell division (Figure S4). These cells exhibited Hes3 immunoreactivity but were not the strongest expressers of Hes3. Other cells with clear vascular association exhibited strong Hes3 expression. These results may indicate that Sox2+ cells that establish association with the vascular niche are better able to maintain Hes3 expression; these cells may also enter a quiescent state. In contrast, fast dividing Sox2+ cells may represent transit amplifying progenitors and they may be in a state of fate commitment.

### CA2 region

Similar results were obtained from the CA2 region of the adult mouse. As in the CA1 region, both Hes3+ and Sox2+ cell numbers increased, with a slightly greater effect on Hes3+ cells. A 1.76 fold increase in Sox2+ cells was measured in the CA2 region greater (Control: 100% +/−5.6, s.e.m.; Treatment: 175.7% +/−9.5, s.e.m.). Again, Hes3+ cell changes were slightly greater (Control: 100% +/−5.4, s.e.m.; Treatment: 208.5% +/−9.5, s.e.m.).

### CA3 region

In the CA3 region, we obtained similar results for Sox2 cell numbers (Control: 100% +/−7.0, s.e.m.; Treatment: 165.5% +/−9.0, s.e.m.). The effect on Hes3+ cell numbers was again greater (Control: 100% +/−8.0, s.e.m.; Treatment: 262.7% +/−16.2, s.e.m.).

### Dentate Gyrus

In the dentate gyrus, the effect of the treatment on both cell populations was less than in the CA3 region. Sox2+ cells showed a significant but slight increase (Control: 100% +/−8.1, s.e.m.; Treatment: 146.9% +/−7.0, s.e.m.). Once again, Hes3+ cells responded more potently than Sox2+ cells (Control: 100% +/−6.8, s.e.m.; Treatment: 183.4% +/−8.7, s.e.m.).

### Hilus

In the hilus we observed relatively weak changes in Sox2+ cell numbers, but potent changes in Hes3+ cells. The increase in Sox2+ cell number was similar to that seen in the dentate gyrus (Control: 100% +/−5.8, s.e.m.; Treatment: 131.1% +/−5.3, s.e.m.). Hes3+ cell number changes were greater (Control: 100% +/−11.5, s.e.m.; Treatment: 265.0% +/−10.8, s.e.m.).

### Hes3 in cultured cells

**Figure 6:**
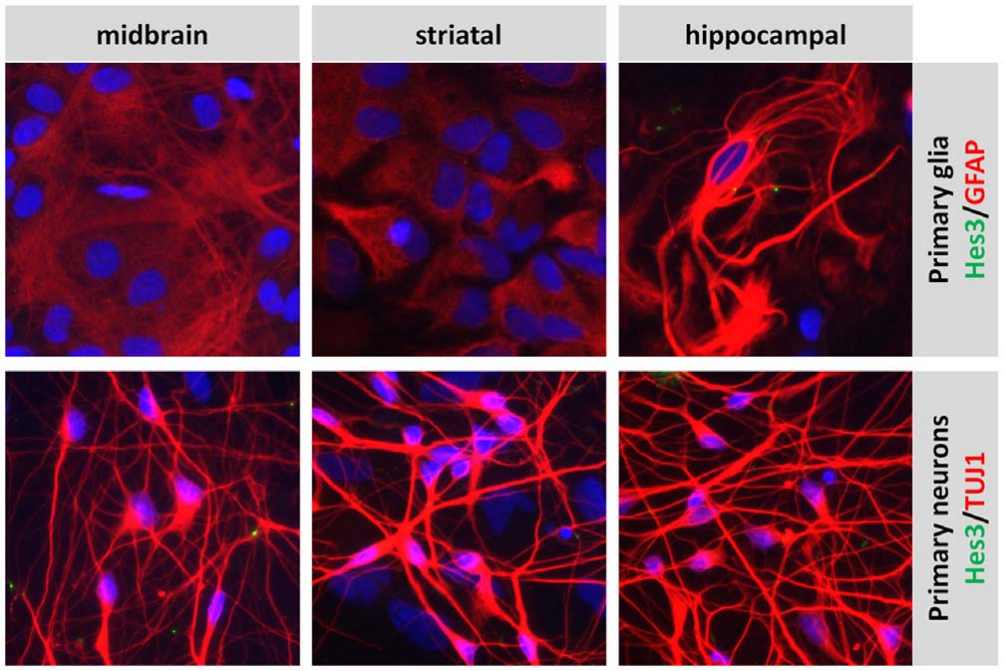
Primary cultures of glia and neurons from the post-natal rat do not express Hes3. Primary cultures of glia and neurons from the rat midbrain, striatum, and hippocampus do not express detectable Hes3. [Width of images: 325 micrometers].

In support of a loss of Hes3 expression in mature glia (identified by immunocytochemistry for the marker Glial Fibrillary Acidic Protein, GFAP) and neurons (identified by immunocytochemistry for the marker class III beta tubulin, using the antibody clone TuJ1), we show that primary cultures of glia and neurons and from the rat midbrain, striatum, and hippocampus exhibit undetectable levels of Hes3 (Figure 6).

## Discussion

In this paper we use a pharmacological approach to increase the numbers of Hes3+ cells in the adult mouse and rat hippocampus. We have previously shown that established fetal neocortex and adult subventricular zone stem cell cultures express Hes3. Hes3 expression is lost when cells are induced to differentiate. In addition, we showed that Hes3+ cells reside in many areas of the adult brain; these can be isolated and their self renewal and multipotential properties can be demonstrated *in vitro* [42], [43], [48], [62], [63]. Hes3+ cell increases correlate with powerful neuroprotective effects, suggesting a role of these endogenous cells in tissue repair. This notion is supported by the fact that Hes3+ cells co-express sonic hedgehog [43], a cytokine with neuroprotective properties. In fact, Hes3 itself regulates sonic hedgehog expression and transfection of Hes3 into mouse fetal neural stem cells induces the expression of sonic hedgehog [42].

Our data suggest the use of this approach in studies of hippocampal function, including plasticity, neurogenesis, regeneration, learning, and memory. The similar results obtained from rats and mice demonstrate the applicability of this approach to experimental models in multiple species. Subtle differences between the two models are discussed below.

Our previous work has established that fetal and adult rodent neural stem cells cultured under standard conditions express Hes3 [42], [43], [62], [63]. Hes3 expression is lost when the cells are induced to differentiate (e.g. by withdrawal of the mitogen basic Fibroblast Growth Factor, bFGF). Hes3+ cells in the adult rat brain typically co-express Sox2, at least under the conditions we have reported [43]. In fact, Hes3 expression marks a subpopulation of Sox2+ cells in many regions of the adult rat brain (approximately 10% of Sox2+ cells in the pharmacologically activated brain co-express Hes3). In the adult brain, Hes3+ cells also co-express the morphogen/mitogen sonic hedgehog (shh) [43]. When cultured fetal mouse neural stem cells are transfected with a plasmid containing the Hes3 gene sequence, expression of shh protein, a mitogen for neural stem cells, increases [42]. In neural stem cell cultures from mice lacking Hes3 expression, the efficacy of Delta4 to stimulate cell survival is largely compromised [48]. These results suggest a role of Hes3 in the maintenance of the endogenous stem cell/progenitor cell population and its usefulness as a biomarker.

In this work we focused our assessments of the effects of the soluble treatments on the number of Hes3+ cells. We did so for a number of reasons. First, the Hes3 gene itself is regulated by the treatments used here [42], [43], [48], [62]. The Notch ligand Delta4, for example, through a non-canonical pathway that includes the fast activation of several kinases (PI3 kinase, Akt, mTOR) and subsequent phosphorylation of STAT3 on the serine residue (but not the tyrosine residue), leads to transcription of the Hes3 gene. Therefore, Hes3+ cell numbers are a measure which is directly relevant to the treatments performed.

Second, Hes3+ cells represent a subpopulation of Sox2+ cells (approximately 10%) [43]. Sox2 is a very useful marker of immature cells in the adult brain but its expression is not confined to these cells. For example, Sox2 expression can be observed in reactive astrocytes in the area of gliosis induced by injury; in contrast, Hes3 is not expressed in areas of gliosis induced by a stab wound (data not shown). Although the percentage of Hes3+ cells that are stem cells (multipotent) or progenitor cells is not yet determined, Hes3 assessments provide a more specific output than the broader, Sox2+ cell population. This may explain the more potent effects observed in the Hes3+/Sox2+ cell population in comparison to the total Sox2+ cell population.

Third, Hes3 measurements offer a distinct advantage: Hes3 expression is maintained even in quiescent states (i.e. non-proliferating), allowing the identification of immature cells in both the active and dormant states. The intracellular localization of Hes3 can vary significantly depending on state. In cultured neural stem cells (under the support of the mitogen bFGF), cells exhibit both nuclear and cytoplasmic Hes3 localization [43], [62]. When bFGF is withdrawn and the differentiation process starts, Hes3 expression becomes exclusively cytoplasmic and eventually disappears altogether. In the unchallenged adult brain, most Hes3+ cells exhibit exclusively cytoplasmic Hes3 localization, whereas cytoplasmic and nuclear Hes3 can be observed when cells from these regions are placed in cultured and stimulated with bFGF [43].

The choice of soluble treatments for this work was based on our previous elucidation of the signals that regulate Hes3 expression and Hes3+ cell numbers. Hes3+ cells express a variety of receptors which render them sensitive to stimuli that regulate Hes3, including Fibroblast Growth Factor receptors, Notch receptors, Tie2 receptors, and the insulin receptor [42], [43], [48], [62], [63]. We have previously shown that the Tie2 receptor is expressed on Sox2+ cells in the hilus of the hippocampus [43], suggesting that the direct target of these soluble factors *in vivo* are the neural precursor cells themselves.

Both experimental paradigms presented here (adult rats treated with a combination of Delta4, Angiopoietin 2, insulin, and a JAK kinase inhibitor; adult mice treated with a combination of Delta4 and Angiopoietin 2) showed fast and potent increases in the number of Hes3+ cells. In addition, in both models we observed the largest effect in the hilus of the dentate gyrus. A quantitative difference between the two paradigms was that in the adult rat, the effects in the hilus were particularly prominent. This difference could be due to species differences, brain size differences, and the fact that in mice we used a simplified combination of treatments. A qualitative difference we observed was that in the rat, Sox2+ cells in the hilus co-expressed Hes3, whereas Sox2+ cells in the subgranular zone did not express Hes3. In contrast, in the mouse, Hes3+/Sox2+ cells are observed throughout the hippocampal areas.

Our results raise the possibility that the cells that provide new granule neurons in the dentate gyrus may be derived from Hes3+ cells in the hilus. We do not present direct data in support of this hypothesis, but the large numbers of Hes3+/Sox2+ cells in the hilus and the lack of Hes3 expression in the rat subgranular zone (the site of neurogenesis, i.e. loss of “stemness”) support this possibility. Future studies may reveal this possibility using appropriate genetic models.

We cannot preclude that subpopulations of Hes3+ cells exist. In fact, adult cultures from the forebrain generate colonies of Hes3+ cells with two distinct morphologies: a short morphology, similar to fetal neural stem cells, and a long morphology, reminiscent of radial glial cells. Indeed, the first are multipotent precursors (they generate neurons, astrocytes, and oligodendrocytes), whereas the latter are confined to the neural lineage, in culture. Ang2 treatment preferentially affects the multipotent Hes3+ cells (it increases their numbers – data not shown). These results support the hypothesis that a multipotent precursor in the hippocampus expresses Hes3 and is the direct target of our pharmacological treatments and future work may be able to conclusively determine this.

Fetal mouse cultures of cortical neural stem cells are highly homogenous and allow for detailed protein phosphorylation analyses by Western Blotting [75]. These cells express Hes3 which is lost when differentiation is induced by the withdrawal of mitogen (basic Fibroblast Growth Factor, bFGF) from the culture medium [42]. These cells also express the receptors for the soluble ligands used here, the Notch, Tie2, and insulin receptors. Activation of the Tie2 receptor by Ang2 and of the Notch receptor by soluble Delta4 activate common downstream kinases, including Akt and STAT3. STAT3 is exclusively phosphorylated on the serine residue, and, subsequently, Hes3 transcriprion is elevated. These results show that pro- and anti-angiogenic factors (Ang2 and Delta4, respectively) activate the same signaling pathway in Hes3+ cells *in vitro* and *in vivo*.

The data also suggest that levels of Hes3 expression may be a useful indicator of the state of these cells. *In vitro*, Hes3+ cells that are induced to differentiate by mitogen withdrawal show a transient increase in Hes3 signal (which may be due to the relocation of Hes3 from the nucleus to the cytoplasm, exhibiting an apparent stronger staining signal). Eventually, Hes3 expression is lost. A similar result was obtained *in vivo*, where particularly strong staining was reported in the inner subgranular zone of the dentate gyrus of the adult mouse hippocampus, the area where neuronal differentiation begins.

We have previously shown that multiple pharmacological means of targeting the endogenous stem cell/progenitor population in the adult brain lead to powerful neuroprotection and behavioral recovery [42], [43], [75]. Here we extend these observations to the hippocampus showing that this brain area can also be heavily modulated in terms of Hes3+ cell numbers. It will be intriguing to extend these studies to models of neurodegenerative disease and stroke affecting the hippocampus, as well as to models used for learning and memory studies, perhaps in combination with higher dose treatments, for example, by the use of repeat injections or osmotic pumps.

In conclusion, our work shows that the use of soluble factors, selected for their ability to activate non-canonical signaling pathways (culminating in the transcription of Hes3) in neural stem cells are capable of inducing powerful increases in the numbers of Hes3+ cells in the adult mouse and rat hippocampus, following single intracerebroventricular injections. It will be of great value to extend these results in order to determine if Hes3+ cells participate in the neurogenic programs of the adult hippocampus and to assess their significance in models of neurodegenerative disease, learning, and memory.

## Materials and Methods

### Ethics Statement

All research involving animals was conducted in accordance with NINDS ACUC (National Institute of Neurological Disorders and Stroke/Animal Care and Use Committee) guidelines and after their approval [Animal Protocol ASP#1204-05]. The animals were socially housed and provided with environmental enrichment and novel food items as provided for by the IACUC-approved NHP enrichment program. Potential postoperative pain was treated with ketoprofen IM, given preemptively at the time of surgery, and for 2 days post-operatively. Prophylactic antibiotic treatment and nursing care were also provided.

### *In vivo* manipulations in adult rats

These experiments were performed as previously described [43]. Treatments (7 Vehicle controls and 7 treated rats) were approved by National Institutes of Health (NIH) guidelines, conforming to the Guide for the care and use of laboratory animals of the U.S. National Institutes of Health (NIH publication 85–23, revised 1996). Male adult (3–6 months) Sprague-Dawley rats (Charles River Laboratories), weighing 250–350 g, were used. Five microliters of different drugs were stereotactically injected into the right lateral ventricle using the following stereotaxic coordinates: Bregma AP −0.9 mm, ML −1.4 mm, VD +3.8 mm. The following reagents were used in combination: Delta4 (2 mg/mL), Ang2 (1 mg/mL), insulin (8 mg/mL), Jak-Inhibitor (20 μM). Animals recovered from the anesthesia and were put back into their home cages, with access to food and water ad libitum. Rats received i.p. injection of the tracer 50 mg/kg BrdU every 12 h for 5 days beginning on day 1 post-op to label dividing cells.

### *In vivo* manipulations in adult mice

Treatments were approved by National Institutes of Health (NIH) guidelines, conforming to the Guide for the care and use of laboratory animals of the U.S. National Institutes of Health (NIH publication 85–23, revised 1996). Experiments were conducted with adult mice (C57BL/6J strain; body weight 18–22 g; The Jackson Laboratory). Eight mice were not injected and served as a baseline. Four were injected in the left lateral ventricle of the brain with saline (NaCl 0.9%) and were used to generate control data. Another two were also injected with saline and inspection showed similar effects. Four mice underwent single injection in the left lateral ventricle of the brain with a combination of Delta4 and Angiopoietin 2 (Two microliters of a combination of: Delta4 (2 mg/mL), Ang2 (1 mg/mL)). Another two mice also underwent single injection in the left lateral ventricle of the brain with a combination of Delta4 and Angiopoietin 2 and inspection confirmed the original data. Injected animals were group housed in standard cages and maintained 7 days in an air-conditioned facility. All animals were euthanized on the 7^th^ day after injection, for immunohistochemical analysis. The experimental procedure was identical both in control and in mice treated with the combination of Delta4 and Angiopoietin 2.

Before surgery, animals were weighed and the appropriate dose for anesthesia was calculated. Mice at postnatal day 66 of age (P66) were anesthetized by injection of a mixture of Rompun (xylazine, 20 mg/ml, 0.5 ml/kg body mass) and Zoletil (tiletamine and zolazepam, 100 mg/ml, 0.5 ml/kg body mass) administered intraperitoneally [76].

Following anesthesia, the fur was shaved from the top of the skull, and the mouse scalp was disinfected with Betadine. The animal was then positioned on the stereotaxic apparatus, the head was fixed using nonrupture ear bars and a 2-cm midsagittal skin incision was made on the scalp in order to visualize the skull landmarker bregma (formed by the cross of the coronal and sagittal sutures). A microdrill was used to perform a small hole (1 mm of diameter) on the left side of the skull according to the previously defined stereotaxic intracerebroventricular coordinates (ICVC): Anteroposterior, AP = −0.1 mm; Mediolateral, ML = +1.0 mm (left side), from bregma. Stereotaxic coordinates were determined from the mouse brain atlas (George Paxinos and Keith B.J. Franklin, Academic Press, 2005). Localization of the final point of injection was previously confirmed by injection of 1 microliter of colorant (Fast Green) in a small subgroup of animals.

A stainless steel guide cannula (Plastics One Inc. –33-gauge, 11.5 mm in length, 0.20 mm in external diameter and 0.10 in internal diameter), was inserted into the hole made previously. After penetrating the dura, we slowly lowered the cannula to the desired Z coordinate of the injection site, and once it reached the right depth [–2 mm dorsoventral (DV) from the dura] slowly (flow speed: 0.15 µl/min), 2 µl of solution (saline or a combination of Delta4 and Angiopoietin 2) were infused into the intracerebroventricular zone of the left brain hemisphere, using a single syringe infusion pump (Harvard Apparatus PHD4400 Programmable Syringe Pump) connected to the cannula via injection tubing previously filled with mineral oil. 2–5 minutes after the end of the infusion we retracted the cannula slowly to avoid backflow of the injected solution to the surface, and removed the animal from the stereotaxic frame.

After cleaning the injection site with sterile saline by moist cotton swabs we sutured the skin with a non-absorbable, sterile, surgical silk suture and disinfected the scalp with Betadine along the incision site. Next, we injected sterile saline solution (30 ml per kilogram body weight) subcutaneously to avoid dehydration of the animal after the surgery, and subsequently we injected the same amount of glucosate solution (5% glucose) to improve the animal feeding immediately after surgical procedure. Finally, we kept the animal warm on a temperature-controlled heating pad (∼37°C) until its full recovery. Once the animal recovered, we returned it to a clean cage and put wet food pellets in the cage for easy access to food.

### Immunohistochemistry

Under deep anesthesia, rats were perfused transcardially with a rinse of saline, followed by 4% formaldehyde fixative (pH 7.4). Brains were removed immediately, stored in the fixative solution overnight, and then in 30% sucrose for 3 days. Brains were frozen-sectioned at 12 or 30 micrometers. Immunohistochemical detection of BrdU was performed with an antigen-retrieval step (sections were boiled in 0.02 M citrate, pH 6.0, in a microwave for 5 min, washed 3 times with PBS, and incubated for 45 min in 2 M HCl, at room temperature).

Wild type mice were deeply anesthetized (by a mixture of Rompun and Zoletil) and transcardially perfused with a saline solution (0.9% NaCl), followed by 4% paraformaldehyde in Phosphate Buffer (PB, pH 7.4). Brains were removed, post-fixed in 4% paraformaldehyde in PB overnight and finally transferred in 30% sucrose in PB for 3 days. Brains were then coronally frozen-sectioned (40 μm thick). Slices were rinsed three times at room temperature (10 min each) in PB, and then blocked in PB with 10% BSA, 0.3% Triton X-1000 for two hours. Sections were then incubated overnight at 4°C in PB with 0.3% Triton X-1000, 0.1% normal donkey serum (NDS) with primary rabbit anti-Hes3 (1∶100 Santa Cruz Biotechnology) and mouse anti-Sox2 (R&D Systems, 1∶100) antibodies. Slices were then rinsed three times in PB (10 min each) at room temperature and incubated with Alexa Fluor 488-conjugated donkey anti-Mouse (1∶350; Jackson IR) and DyLight 594-conjugated donkey anti-Rabbit (1∶350; Jackson IR) secondary antibodies for 3.5 hrs at room temperature. Slices were rinsed three times in PB (10 min each) at room temperature and coverslipped in mounting medium. Immunofluorescence was then observed with a laser confocal microscope (Leica SP5) and images were acquired.

### Quantifications

The number of animals per experimental group is given in the text (Results section). Rat hippocampal sections were collected between bregma +3.14 and +5.3 mm. Mouse hippocampal sections were collected between bregma +1.58 and −2.46 mm.

### Statistical analysis

Results shown are means +/− SEM. Asterisks identify experimental groups that were significantly different (p value <0.05) from control groups by the Student's t test (Microsoft Excel), where applicable.

### Reagents

We used the following reagents and antibodies: mouse Delta4 (1389-D4), human Angiopoietin-2 (623-AN), from R&D Systems; BrdU (84447723) from Boehringer; Alexa-Fluor-conjugated secondary antibodies from Invitrogen; DAPI (D-8417) from Sigma, and general chemicals from Sigma. For immunohistochemical staining, we used antibodies against the following markers: BrdU (H5903; Accurate), Sox2 (MAB2018; R&D Systems); Hes3 (25393) from Santa Cruz Biotechnology.

## Supporting Information

Figure S1 Soluble factors increase the numbers of Hes3+ cells in the adult mouse dentate gyrus and hilus. Representative images show Sox2+ and Hes3+ cells in various areas of the control (saline-injected) hippocampus. [Width of images: 115 micrometers].
(TIF)

Figure S2 Hes3+ cells in the adult mouse dentate gyrus of the hippocampus. Hes3+ cells in the adult mouse hippocampus in the control and pharmacologically activated hippocampus. [Width of images: 750 micrometers].
(TIF)

Figure S3 Sox2+/Hes3+ cells in the adult mouse CA3 region of the hippocampus. Sox2+/Hes3+ cells in the adult mouse hippocampus in the control and pharmacologically activated hippocampus. [Width of images: 375 micrometers].
(TIF)

Figure S4 Sox2+/Hes3+ cells in the CA3 region of the activated adult hippocampus. Sox2+/Hes3+ cells in the adult mouse CA3 region of treated mice often appear in pairs. [Width of images: top: 115 micrometers; bottom: 80 micrometers].
(TIF)

Table S1 Cell number per 100 micrometer squared in 40 micrometer thick sections. The table presents the number of Sox2+ and Hes3+ cells in different regions of the adult mouse hippocampus in control (saline-injected) and experimental mice (injected with a combination of Delta4 and Ang2). Values are absolute numbers (numbers of cells per 100 micrometers squared, in brain sections of 40 micrometer thickness).
(DOCX)

Table S2 Fold increases in cell number by Delta4+Ang2. The table presents fold changes in the numbers of Sox2+ and Hes3+ cells in different areas of the adult mouse hippocampus following pharmacological treatment with a combination of Delta4 and Ang2.
(DOCX)
