# Supplementary figures and images for: Fast, Potent Pharmacological Expansion of Endogenous Hes3+/Sox2+ Cells in the Adult Mouse and Rat Hippocampus
Source: PLoS One. 2012 Dec 10;7(12):e51630. doi: 10.1371/journal.pone.0051630 (PMC3518467; doi:10.1371/journal.pone.0051630)

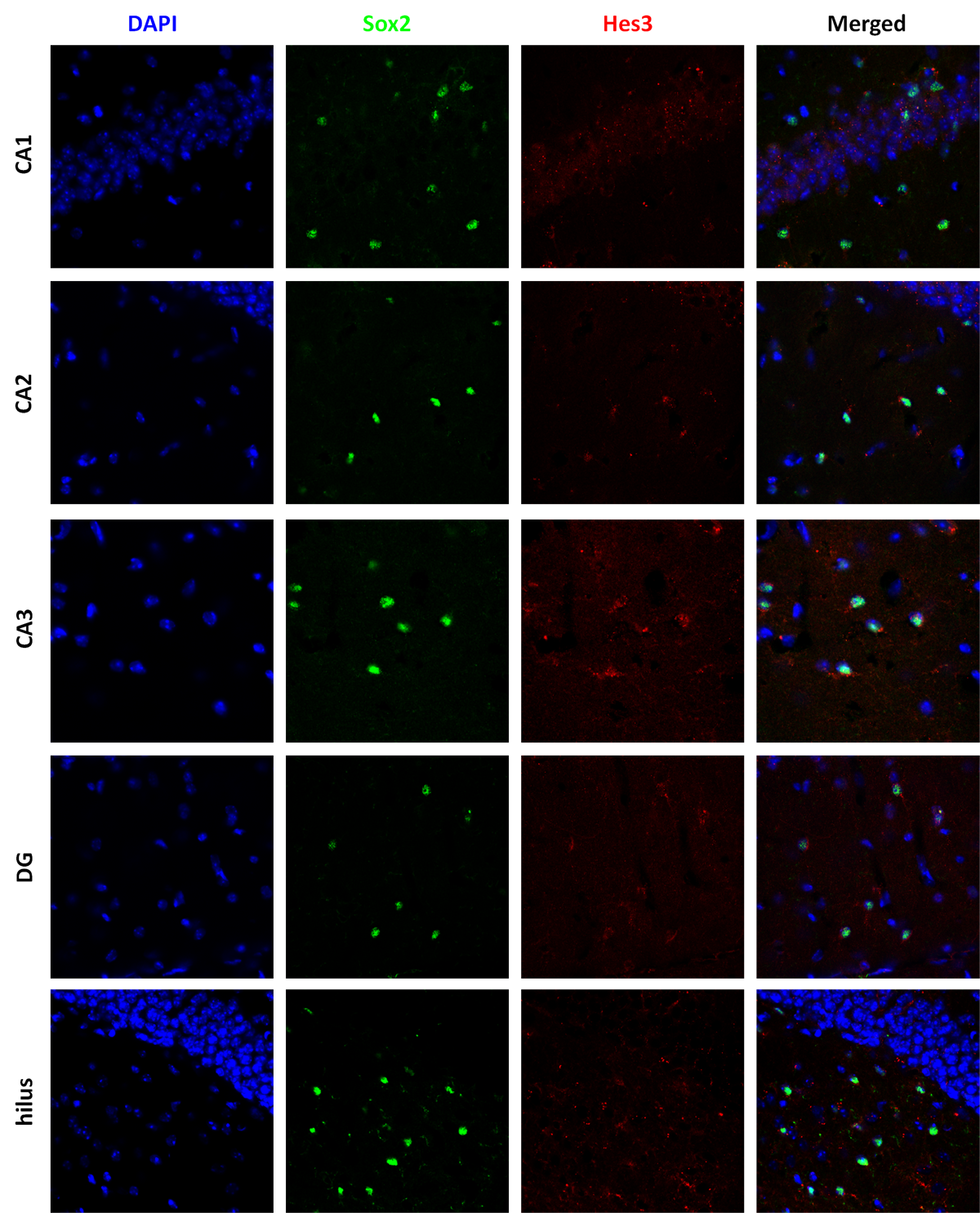

Supplement: Figure S1 — Soluble factors increase the numbers of Hes3+ cells in the adult mouse dentate gyrus and hilus. Representative images show Sox2+ and Hes3+ cells in various areas of the control (saline-injected) hippocampus. [Width of images: 115 micrometers]. (TIF) [file pone.0051630.s001.tif]

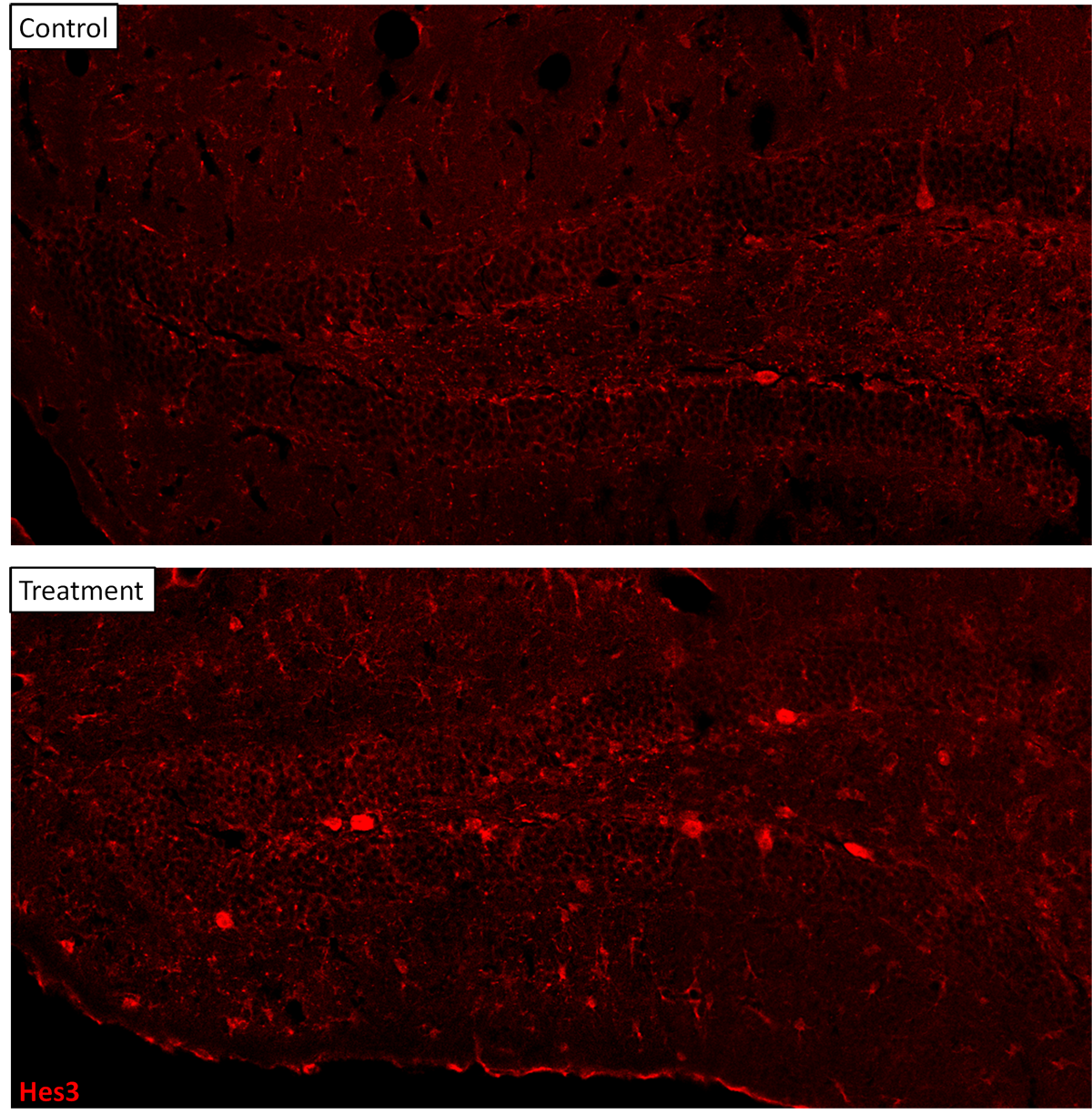

Supplement: Figure S2 — Hes3+ cells in the adult mouse dentate gyrus of the hippocampus. Hes3+ cells in the adult mouse hippocampus in the control and pharmacologically activated hippocampus. [Width of images: 750 micrometers]. (TIF) [file pone.0051630.s002.tif]

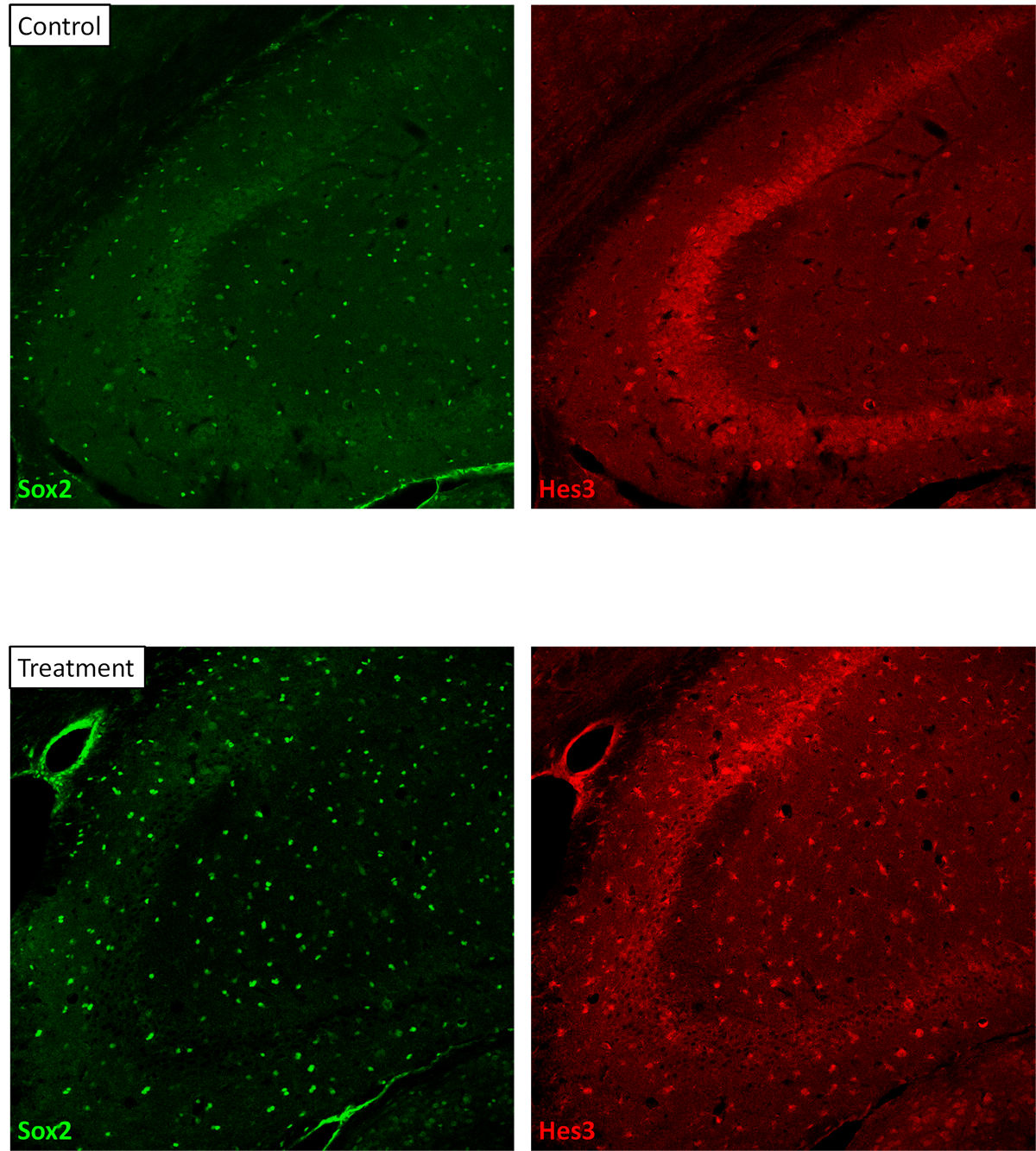

Supplement: Figure S3 — Sox2+/Hes3+ cells in the adult mouse CA3 region of the hippocampus. Sox2+/Hes3+ cells in the adult mouse hippocampus in the control and pharmacologically activated hippocampus. [Width of images: 375 micrometers]. (TIF) [file pone.0051630.s003.tif]

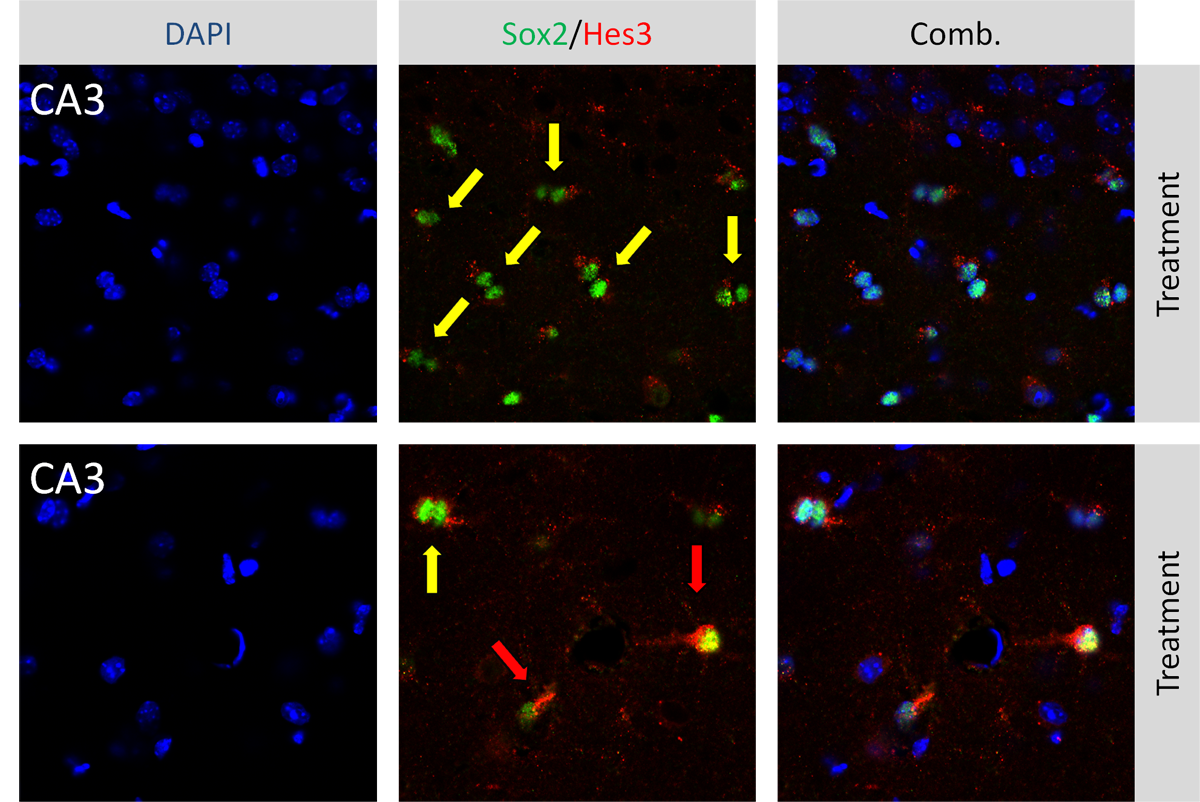

Supplement: Figure S4 — Sox2+/Hes3+ cells in the CA3 region of the activated adult hippocampus. Sox2+/Hes3+ cells in the adult mouse CA3 region of treated mice often appear in pairs. [Width of images: top: 115 micrometers; bottom: 80 micrometers]. (TIF) [file pone.0051630.s004.tif]
