# Supplementary material for: Fast, Potent Pharmacological Expansion of Endogenous Hes3+/Sox2+ Cells in the Adult Mouse and Rat Hippocampus
Source: PLoS One. 2012 Dec 10;7(12):e51630. doi: 10.1371/journal.pone.0051630 (PMC3518467; doi:10.1371/journal.pone.0051630)
Supplement: Table S1 — Cell number per 100 micrometer squared in 40 micrometer thick sections. The table presents the number of Sox2+ and Hes3+ cells in different regions of the adult mouse hippocampus in control (saline-injected) and experimental mice (injected with a combination of Delta4 and Ang2). Values are absolute numbers (numbers of cells per 100 micrometers squared, in brain sections of 40 micrometer thickness). (DOCX) [file pone.0051630.s005.docx]

| **Biomarker** | **Treatment** | **Area** | **Cells/100um2** | **SD** | **sem** | **p** |
| --- | --- | --- | --- | --- | --- | --- |
|  |  |  |  |  |  |  |
| **Sox2+ cells** | **Control** | **CA1** | 7.75 | 2.40 | 0.67 | <0.001 |
|  |  | **CA2** | 7.96 | 1.61 | 0.45 | <0.001 |
|  |  | **CA3** | 6.64 | 1.67 | 0.46 | <0.001 |
|  |  | **DG** | 6.91 | 2.04 | 2.02 | <0.001 |
|  |  | **hilus** | 9.66 | 2.02 | 0.56 | <0.001 |
|  |  |  |  |  |  |  |
|  | **Delta4+Ang2** | **CA1** | 12.86 | 2.41 | 0.47 | <0.001 |
|  |  | **CA2** | 13.98 | 3.72 | 0.76 | <0.001 |
|  |  | **CA3** | 11.00 | 3.12 | 0.60 | <0.001 |
|  |  | **DG** | 10.14 | 2.45 | 0.48 | <0.001 |
|  |  | **hilus** | 12.67 | 2.57 | 0.51 | <0.005 |
|  |  |  |  |  |  |  |
| **Hes3+ cells** | **Control** | **CA1** | 6.71 | 2.36 | 0.65 | <0.001 |
|  |  | **CA2** | 6.64 | 1.29 | 0.36 | <0.001 |
|  |  | **CA3** | 4.30 | 1.24 | 0.34 | <0.001 |
|  |  | **DG** | 5.92 | 1.44 | 0.40 | <0.001 |
|  |  | **hilus** | 4.50 | 1.87 | 0.52 | <0.001 |
|  |  |  |  |  |  |  |
|  | **Delta4+Ang2** | **CA1** | 13.67 | 2.67 | 0.52 | <0.001 |
|  |  | **CA2** | 13.85 | 3.09 | 0.63 | <0.001 |
|  |  | **CA3** | 11.29 | 3.62 | 0.70 | <0.001 |
|  |  | **DG** | 10.85 | 2.28 | 0.51 | <0.001 |
|  |  | **hilus** | 11.91 | 3.31 | 0.49 | <0.001 |
