# Supplementary material for: Fast, Potent Pharmacological Expansion of Endogenous Hes3+/Sox2+ Cells in the Adult Mouse and Rat Hippocampus
Source: PLoS One. 2012 Dec 10;7(12):e51630. doi: 10.1371/journal.pone.0051630 (PMC3518467; doi:10.1371/journal.pone.0051630)
Supplement: Table S2 — Fold increases in cell number by Delta4+Ang2. The table presents fold changes in the numbers of Sox2+ and Hes3+ cells in different areas of the adult mouse hippocampus following pharmacological treatment with a combination of Delta4 and Ang2. (DOCX) [file pone.0051630.s006.docx]

| **Biomarker** | **Area** | **Fold change** |
| --- | --- | --- |
|  |  |  |
| **Sox2+ cells** | **CA1** | 1.66 |
|  | **CA2** | 1.76 |
|  | **CA3** | 1.66 |
|  | **DG** | 1.47 |
|  | **hilus** | 1.31 |
|  |  |  |
| **Hes3+ cells** | **CA1** | 2.04 |
|  | **CA2** | 2.09 |
|  | **CA3** | 2.63 |
|  | **DG** | 1.83 |
|  | **hilus** | 2.65 |
